# Merkel Cell Carcinoma With Lymph Node Metastasis: A Case Emphasizing Early Diagnosis and Multidisciplinary Management

**DOI:** 10.7759/cureus.90232

**Published:** 2025-08-16

**Authors:** Azalea Guadalupe Altamirano De La Cruz, Christian Daniel Orozco Velazco, Abraham Ramírez Saavedra, Maria Daniela Salazar López, Karen Sofia Cruz Dominguez

**Affiliations:** 1 Internal Medicine, Mexican Social Security Institute (IMSS) General Zone Hospital No. 8, Ensenada, MEX; 2 Internal Medicine, Hospital Angeles Metropolitano, Mexico City, MEX; 3 Medical Science, Universidad de Guanajuato, Guanajuato, MEX; 4 Internal Medicine, Institute for Social Security and Services for State Workers HG90, La Paz, MEX

**Keywords:** ck20, elderly patient case report, immunohistochemistry (ihc), ki-67 proliferation index, merkel cell carcinoma (mcc)

## Abstract

Merkel cell carcinoma (MCC) is a rare but exceptionally aggressive neuroendocrine skin cancer, characterized by low global incidence yet significant clinical impact due to its propensity for early metastasis. Regional lymph node metastases occur frequently, while distant metastases are present in a substantial proportion of patients. The rising incidence over recent decades is largely attributed to the aging global population, associated immunosenescence, and increased cumulative ultraviolet (UV) radiation exposure, particularly UVB radiation.

MCC’s medical significance lies in its rapid progression, high metastatic potential, and poor prognosis, with survival rates decreasing markedly from localized disease to cases with regional lymph node involvement. This underscores the need for prompt recognition and management to optimize patient outcomes. Key risk factors include advanced age, with an exponential increase in incidence after retirement age, immunosuppression, and chronic sun/UV exposure. Merkel cell polyomavirus has been identified as an important etiologic factor in the majority of cases.

This case report describes an elderly female patient who presented with a rapidly growing, ulcerated cervical lesion that had progressed substantially over several months. The lesion was initially misdiagnosed and inadequately treated with minor excision without histopathological analysis. Subsequent rapid local recurrence, extensive regional lymph node involvement, and definitive diagnosis, confirmed by characteristic immunohistochemical staining patterns, highlight the critical need for high clinical suspicion and meticulous histopathological evaluation.

Palliative radiotherapy provided measurable clinical benefit, including significant pain relief and notable tumor size reduction. This case exemplifies how deficiencies in initial management can dramatically alter disease trajectory in aggressive malignancies, transforming potentially curable early-stage disease into advanced locoregional involvement requiring palliative treatment with markedly reduced survival expectations.

## Introduction

Merkel cell carcinoma (MCC) stands as one of the most challenging cutaneous neoplasms in contemporary oncological practice, representing a rare but exceptionally aggressive neuroendocrine skin cancer that demands immediate attention from clinicians and pathologists specializing in dermatologic oncology. This case report aims to illustrate the devastating consequences of delayed MCC diagnosis and emphasize the critical importance of systematic histopathological analysis of all cutaneous lesions, regardless of their apparent benign nature.

Clinically, MCC typically presents as a red-purple, firm, rapidly growing nodule in sun-exposed areas, particularly the head, neck, and extremities. This aggressive skin cancer demonstrates accelerated growth patterns (often >1 cm in <3 months), frequently progressing to ulceration while remaining characteristically painless in most cases, though approximately 30% of patients may experience discomfort.

Despite comprising less than 1% of all skin cancers, this rare and exceptionally aggressive neuroendocrine entity possesses unique clinical and biological characteristics that significantly distinguish it from other cutaneous malignancies [1]. Its importance extends beyond its low frequency, as its highly aggressive clinical behavior and capacity for early metastasis make it a major concern for clinicians and pathologists specializing in dermatologic oncology. Early recognition is critical due to its tendency to be initially misdiagnosed as benign lesions such as sebaceous cysts, lipomas, or basal cell carcinomas, a diagnostic pitfall that can have devastating consequences, as illustrated in this case.

The epidemiology of MCC reveals a complex and evolving landscape, with a global incidence ranging between 0.1 and 1.6 cases per 100,000 person-years, and Australia reporting the highest rates worldwide at 1.6 cases per 100,000 [2]. This particular geographical distribution suggests a strong correlation with environmental factors, especially intense and prolonged solar exposure. The documented increase in incidence over recent decades is a multifactorial phenomenon, reflecting not only global population aging with associated immunosenescence (age-related decline of the immune system) but also shifts in recreational and occupational sun exposure patterns, as well as advancements in diagnostic techniques that have facilitated better identification of this previously underdiagnosed entity [3].

The pathogenesis of MCC involves a complex interplay between intrinsic host factors and specific environmental exposures. Among established risk factors, advanced age consistently emerges as the most significant predictor, with incidence increasing exponentially after 65 years, a phenomenon closely linked to immunosenescence and diminished immune surveillance capacity [4]. Immunosuppressed states, whether congenital or acquired, significantly elevate the risk of MCC development, with a 5-10-fold increased risk particularly relevant in solid organ transplant recipients, HIV-infected patients, or those undergoing chronic immunosuppressive therapy.

The discovery of Merkel Cell Polyomavirus (MCPyV) as a fundamental etiologic factor has revolutionized our understanding of this neoplasm, identified as responsible for approximately 80% of diagnosed cases [5]. MCPyV oncoproteins (viral proteins that drive cellular transformation) represent key molecular drivers in MCPyV-positive cases. Clonal integration of MCPyV into the cellular genome represents an early event in carcinogenesis, suggesting that neoplastic transformation is a process dependent on viral persistence and the continuous expression of viral oncoproteins. MCPyV-negative cases (~20%) typically arise through UV-induced mutations in tumor suppressor genes, explaining the strong association with sun exposure in this subset [6,7].

The clinical behavior of MCC is characterized by exceptional aggressiveness, distinguishing it from most cutaneous neoplasms. Local recurrence rates range from 25-30%, while regional lymph node metastases occur in 50-70% of cases, and distant metastases in 35-50% of patients [8]. This propensity for early metastasis reflects the tumor's biologically aggressive nature and underscores the critical importance of early diagnosis and precise staging. The rapidity with which MCC can progress from an apparently benign lesion to systemic metastatic disease represents one of the greatest challenges in its clinical management.

The stage at diagnosis constitutes the most determinant prognostic factor in MCC, with regional nodal involvement being the primary predictor not only of distant metastatic disease but also of overall survival, where five-year survival rates decrease dramatically from 75% in localized disease to 35% with regional lymph node involvement [9]. This stage-to-prognosis relationship is particularly pronounced in MCC compared to other cutaneous neoplasms, where the presence of microscopic nodal metastases can dramatically alter the clinical course and available therapeutic options.

Clinical warning signs that should raise immediate suspicion for MCC include rapid growth with significant size increase over weeks to months, characteristic red-purple discoloration, firm consistency, occurrence in elderly patients with chronic sun exposure, and location in high-risk anatomical sites such as the head, neck, and upper extremities. The absence of pain should not provide false reassurance, as most MCCs are painless initially. Any combination of these factors should trigger immediate biopsy and specialist consultation.

The diagnostic approach to MCC requires a sophisticated combination of histopathological evaluation and immunohistochemistry, where characteristic cellular morphology must be complemented by a panel of specific markers to confirm the differential diagnosis [10]. The demonstration of positivity for neuroendocrine markers, with INSM1 (insulinoma-associated protein 1) being the most sensitive of the neuroendocrine markers according to the WHO Blue Book [11,12], along with chromogranin, synaptophysin, CD56, and the distinctive and virtually pathognomonic paranuclear staining pattern of cytokeratin 20 (CK20), forms the cornerstone of definitive diagnosis [12].

The neuroendocrine nature of MCC has direct clinical implications: it determines specific therapeutic options, including targeted treatments, influences prognostic stratification, and guides the selection of appropriate chemotherapy regimens when systemic treatment is indicated. Understanding these molecular characteristics enables clinicians to make informed therapeutic decisions and provide accurate prognostic information to patients.

The ascending epidemiological trend observed in recent decades reflects multiple converging factors, including global population aging with its associated immunosenescence, increased cumulative ultraviolet radiation exposure due to changes in lifestyle and recreational patterns, and significant improvements in diagnostic techniques and clinical awareness of this entity [13]. This increase in reported incidence may also reflect better immunohistochemical characterization that allows for the distinction of MCC from other neuroendocrine neoplasms.

The diagnostic complexity of MCC underscores the importance of a multidisciplinary approach involving not only specialized pathological expertise but also coordination among dermatologists, medical oncologists, radiation oncologists, and surgeons specializing in cutaneous cancer [14]. The aggressive nature of the tumor, combined with its variable clinical presentation, makes early recognition and appropriate management fundamental to optimizing therapeutic outcomes and improving the overall survival of affected patients.

## Case presentation

A 76-year-old female patient presented for medical consultation with a significant medical history of arterial hypertension controlled with angiotensin-converting enzyme inhibitors and type 2 diabetes mellitus managed with metformin. Initial functional status assessment revealed ECOG Performance Status 1, which subsequently progressed to ECOG 3 at treatment initiation due to disease burden. Importantly, the patient did not present with known immunosuppressive conditions and was not receiving immunosuppressive treatments.

Regarding risk factors, the patient reported a history of moderate recreational sun exposure typical for her age group and domestic activities, with estimated cumulative lifetime sun exposure consistent with her rural background. Medical documentation revealed no history of immunosuppressive medications, organ transplantation, or HIV infection. Immune status assessment revealed no evidence of primary or secondary immunodeficiency, with no history of organ transplantation, HIV infection, chronic immunosuppressive therapy, or hematologic malignancies, factors that are particularly relevant given MCC's strong association with immunosuppression.

Clinical history and detailed chronology

The patient reported the initial appearance of a nodular lesion in the cervical region that had been previously treated with minor surgical excision without histopathological analysis of the removed specimen. This case exemplifies the devastating consequences of inadequate initial management, where failure to perform histopathological analysis of the original excision specimen resulted in a critical three-month diagnostic delay during which the tumor progressed from potentially localized disease to extensive regional involvement.

The specific chronology of events developed as follows: in January 2022, the initial appearance of the cervical nodular lesion measuring approximately 1 cm occurred. Subsequently, in February 2022, a minor surgical excision was performed by the primary care physician without histopathological analysis of the specimen. Recurrence was observed in June 2022 with rapid growth and ulceration development. Medical consultation and incisional biopsy were performed on October 5, 2022, followed by histopathological confirmation of MCC with immunohistochemistry on October 20, 2022. CT staging was completed on October 25, 2022, with a multidisciplinary tumor board decision made on October 30, 2022, and initiation of palliative radiotherapy on November 15, 2022.

Approximately three months after the initial excision, the patient observed with concern the appearance of a new lesion at the same anatomical site. This new lesion was characterized by progressive and accelerated growth that alarmed both the patient and her family members. The lesion exhibited significant morphological changes, including superficial ulceration, notable color changes, and considerable size increase over a relatively short period, progressing from initial appearance to a 10 cm diameter in approximately four months.

Physical examination

**Figure 1:**
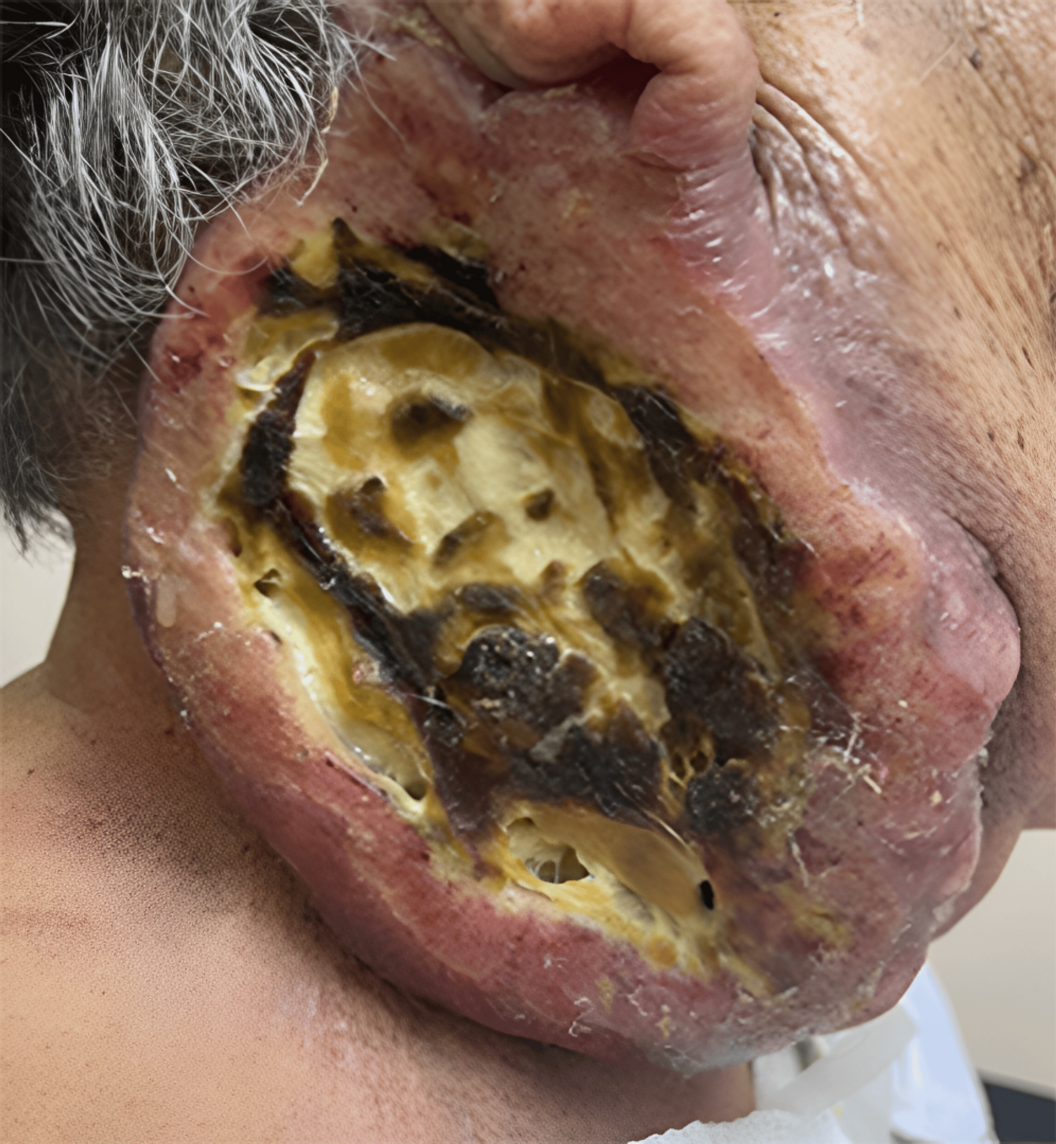
Extensive ulcerated retroauricular tumor (10 cm) with central necrosis and deep tissue infiltration.

At the time of consultation, the patient presented with stable vital signs and no evidence of systemic compromise. Physical examination revealed a primary lesion in the right lateral cervical region with tomographic dimensions of 9.0 × 7.0 × 6.5 cm and a maximum clinical diameter of approximately 10 cm. The lesion demonstrated distinctive morphological characteristics, including an ulcerated surface with irregular and elevated borders, firm consistency on palpation, and erythematous coloration with characteristic violaceous areas (Figure 1).

Lymph node assessment through physical examination revealed non-palpable superficial cervical lymphadenopathy, although a significant clinical-radiological discrepancy was noted due to the patient's particular anatomical characteristics, including age-related body habitus, short neck anatomy, and abundant cervical adipose tissue that limited adequate palpation of deep lymph node chains.

The patient reported no pain associated with the lesion but expressed marked concern about the aesthetic appearance and rapid growth she had observed. During history-taking, no systemic symptoms such as unintentional weight loss, significant fatigue, febrile episodes, or night sweats were documented. The patient denied significant occupational sun exposure, though she reported recreational sun exposure typical for her age and routine domestic activities.

Histopathological analysis

Microscopic analysis revealed a neoplastic proliferation composed of small cells with hyperchromatic nuclei and scant cytoplasm, showing a diffuse growth pattern with characteristic solid architecture. The cells exhibited high-grade nuclear atypia, elevated mitotic activity, and areas of tumor necrosis, findings that together were consistent with small cell neuroendocrine carcinoma.

Specific immunohistochemistry techniques

Immunohistochemical analysis was performed using an automated immunostainer with standard protocols. Antigen retrieval was achieved through the heat-induced epitope retrieval (HIER) method, followed by primary antibody incubation for 60 minutes at room temperature. Detection was accomplished using a polymer-based detection system with DAB chromogen.

**Figure 2:**
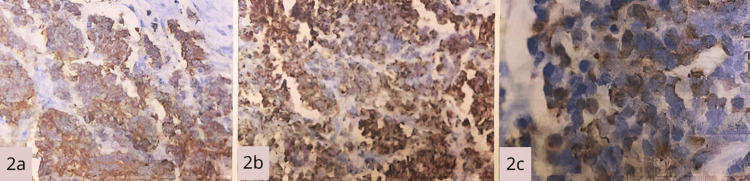
(a) Positive CD56 immunohistochemistry (neuroendocrine marker), 400×, demonstrating diffuse cytoplasmic staining confirming neuroendocrine differentiation of tumor cells. (b) Diffuse positive chromogranin A immunostaining, 400×, confirming neuroendocrine differentiation. Strong, diffuse cytoplasmic positivity throughout tumor cells is consistent with a neuroendocrine neoplasm. (c) CK20 immunostaining showing pathognomonic paranuclear dot pattern, 400×, exhibiting the characteristic perinuclear dotted staining pattern diagnostic of Merkel cell carcinoma. This distinctive paranuclear "dot-like" immunoreactivity is pathognomonic for MCC and distinguishes it from other small round blue cell tumors. Insert: High-power view demonstrating typical paranuclear dots.

Quantitative immunohistochemistry results demonstrated highly specific diagnostic findings. CK20 showed positive staining with the characteristic paranuclear pattern, highly specific for MCC. CD56 demonstrated diffusely positive cytoplasmic staining, indicating neuroendocrine differentiation. Chromogranin A exhibited diffusely positive, strong cytoplasmic staining, confirming neuroendocrine differentiation. Synaptophysin demonstrated diffusely positive cytoplasmic staining as an additional neuroendocrine marker. Ki-67 showed 80% positive nuclei, indicating a high proliferation index with significant prognostic implications (Figure 2).

Evaluation criteria

Positive staining was defined as greater than 10% of tumor cells showing the characteristic pattern for each respective marker. The Ki-67 proliferation index was calculated by counting 1000 tumor cell nuclei in hotspot areas under high magnification. The CK20 paranuclear pattern was considered pathognomonic when present in greater than 50% of tumor cells with the distinctive perinuclear dotted appearance (Table 1).

**Table 1:** Quantitative IHC results. IHC: immunohistochemistry, MCC: Merkel cell carcinoma.

Marker	Result	Pattern	Interpretation
CK20	Positive	Paranuclear dot pattern	Pathognomonic for MCC
CD56	Diffusely positive	Cytoplasmic staining	Neuroendocrine differentiation
Chromogranin A	Diffusely positive	Strong cytoplasmic	Neuroendocrine confirmation
Synaptophysin	Diffusely positive	Cytoplasmic	Neuroendocrine marker
Ki-67	80% positive nuclei	Nuclear staining	High proliferation index

Radiological evaluation

Staging evaluation was performed using intravenous contrast-enhanced computed tomography of the head and neck with 2.5 mm slice thickness and 100 mL of iodinated contrast agent. Image analysis included axial, coronal, and sagittal reconstructions for comprehensive anatomical evaluation.

Imaging findings revealed a right lateral cervical tumor mass measuring 9 × 7 × 5 cm with deep muscle invasion involving the paravertebral musculature. The tumor demonstrated extension into deep cervical spaces with contact to cervical vascular structures without clear invasion. Notably, no cortical bone destruction or osseous involvement was identified despite extensive soft tissue infiltration.

Extensive nodal involvement was documented with metastases identified at all cervical levels (I-VI) and ipsilateral supraclavicular chain involvement. Multiple enlarged lymph nodes were present, with the largest measuring 2 cm, and extracapsular extension was documented in several nodes. More than 10 positive lymph nodes were confirmed, meeting criteria for N3 classification.

**Figure 3:**
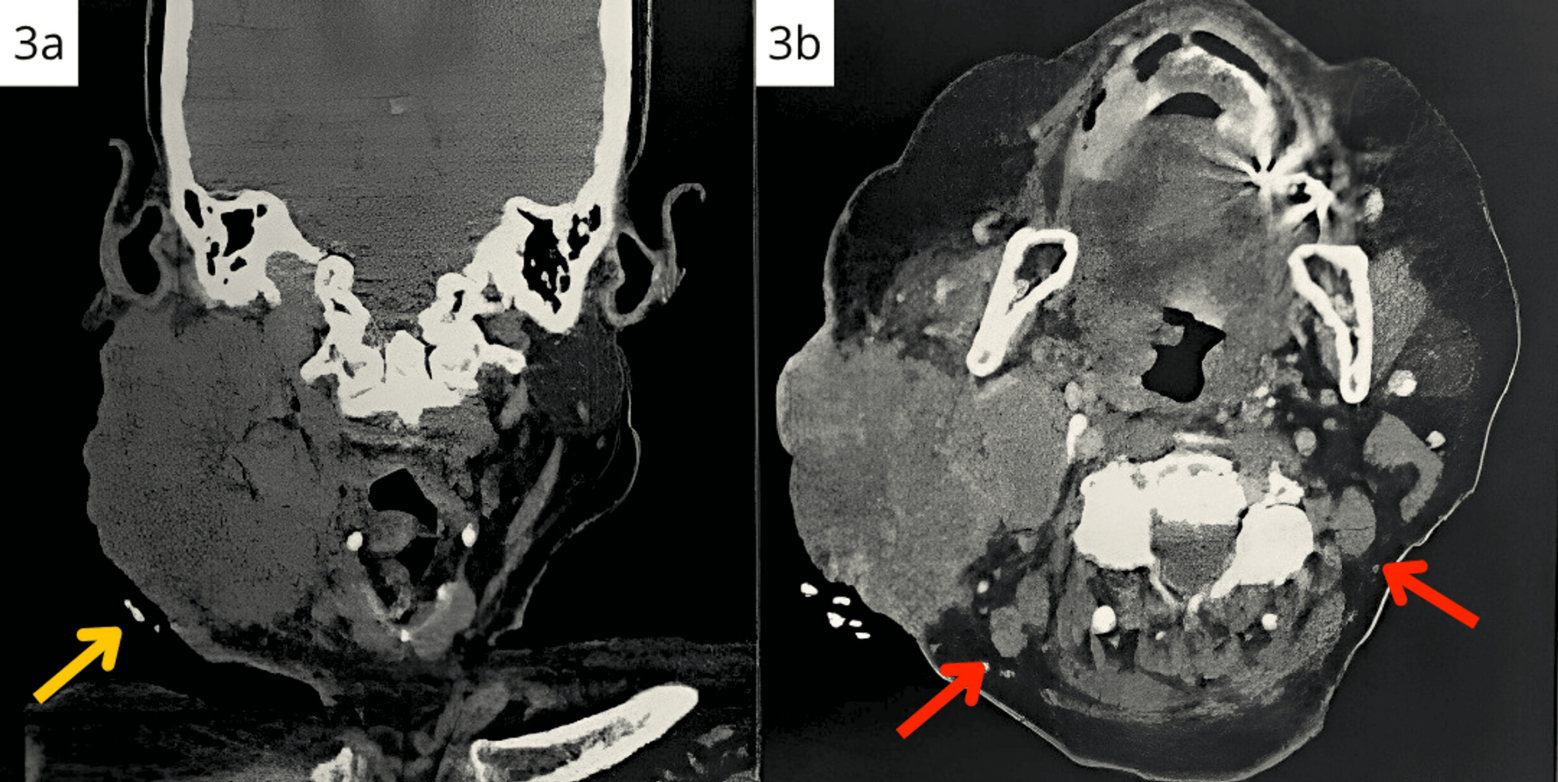
(a) CT coronal image (yellow arrow). Large ill-defined solid tumor in the right neck (9 × 7 × 6.5 cm) with cystic/necrotic components and intralesional vessels. The mass invades the skin, parotid and submandibular glands, masticator and paraspinal muscles, and the retromandibular trigone. It compresses the right internal jugular vein. Infiltrative ipsilateral parotid and right supraclavicular lymphadenopathy are present. No cortical bone destruction or osseous invasion is identified despite extensive soft tissue infiltration of adjacent muscular structures. (b) CT transversal image (red arrow). Infiltrative lymph nodes, up to 20 mm, are observed in the peritumoral and residual ipsilateral parotid gland regions (approximate maximum clinical diameter: 10 cm).

Comprehensive evaluation revealed no evidence of pulmonary, hepatic, or other distant organ metastases on available imaging studies. Final staging was determined as T4N3M0 based on deep muscular invasion (T4), extensive regional lymph node involvement with greater than 10 positive nodes and extracapsular extension (N3), and absence of distant metastases (M0) (Figure 3).

Multidisciplinary team decision

The case was presented to a comprehensive multidisciplinary tumor board with participation from radiation oncology, medical oncology, and surgical oncology specialists. Considering the advanced disease stage (T4N3M0), the patient's advanced age, and deteriorated performance status (ECOG 3), a palliative treatment approach was determined to be the most appropriate therapeutic strategy.

Technical radiotherapy specifications

A hypofractionated radiotherapy regimen with palliative intent was designed, consisting of a total dose of 30 Gy delivered in six fractions of 5 Gy each. The treatment utilized the IMRT (intensity-modulated radiation therapy) technique with bilateral cervical fields encompassing both the primary tumor and involved nodal regions. The treatment schedule consisted of three fractions per week delivered over two weeks.

Primary treatment objectives included control of tumor growth-associated pain, reduction of tumor burden for local symptom relief, and prevention of locoregional complications such as progressive ulceration and bleeding.

Quantitative treatment response assessment

Treatment response was assessed using multiple standardized evaluation methods, including the visual analog scale (VAS) for pain assessment on a 0-10 scale, ECOG Performance Status monitoring for functional assessment, and clinical evaluation of local symptoms, including ulceration control and tumor-related secretions.

Measured treatment outcomes demonstrated clinical benefits across multiple parameters. Pain control assessment showed significant improvement, with VAS scores decreasing from 8/10 pre-treatment to 3/10 post-treatment, representing a 62.5% reduction in pain intensity. ECOG Performance Status remained stable at level 3 without further deterioration, indicating maintenance of baseline functional capacity. Local symptom control improved significantly, with better ulceration control and reduction of tumor-related secretions.

Treatment tolerance and supportive care

The patient successfully completed the full radiotherapy regimen with an acceptable tolerance profile. No grade 3-4 acute toxicities were observed during treatment delivery, and the patient maintained baseline functional status without further deterioration throughout the treatment period.

Comprehensive supportive care was implemented, including a multimodal analgesic protocol incorporating both opioid and non-opioid agents for optimal pain management. Specialized wound care protocols were established with infection prevention measures to address the ulcerative component. Nutritional support included dietary consultation and appropriate supplementation to maintain adequate nutritional status. Psycho-oncological support encompassed mental health assessment and ongoing psychological support throughout the treatment period.

## Discussion

MCC represents one of the most complex and challenging cutaneous malignancies in contemporary oncological practice, constituting a dermal neuroendocrine entity whose optimal management and prognostic characterization remain active areas of research in specialized medical literature [11]. The intrinsic rarity of this neoplasm, combined with its biologically aggressive behavior and capacity for rapid progression to advanced stages, poses multidimensional challenges ranging from early clinical recognition to the implementation of effective, evidence-based therapeutic strategies.

While population-based data demonstrate that 65% of MCC patients present with localized disease at diagnosis, 26% with regional lymph node involvement, and only 8% with distant metastases [14], our case represents a particularly challenging clinical scenario. The extensive T4N3M0 presentation, with deep muscular invasion (masticator and paraspinal muscles) and extensive regional lymphadenopathy across multiple cervical levels, places this patient within the most advanced subset of locoregionally confined disease. This clinical presentation illustrates the dramatic prognostic implications of diagnostic delay, where progression from potentially localized disease to N3 stage results in five-year survival rates decreasing from 75% to 35% [15]. This progression underscores several critical clinical learning points: (1) the importance of comprehensive staging workup even in elderly patients, (2) the multidisciplinary approach required for advanced locoregional disease, and (3) the shift toward palliative strategies when curative resection is not feasible due to extensive local invasion and patient factors.

The clinical recognition of MCC presents distinctive characteristics that, although not pathognomonic, constitute important semiotic elements for differential diagnosis. The typical presentation includes the appearance of a rapidly growing nodule, generally ranging from 0.5 to 5 cm in diameter, which in its initial stages tends to be painless, presenting a characteristic firm consistency and a distinctive discoloration varying from erythematous tones to red-purple hues that can evolve into ulceration in cases of advanced disease [15]. This rapid morphological progression represents one of the most alarming clinical characteristics of MCC and frequently constitutes the reason for consultation, leading to specialized medical evaluation.

This topographic distribution consistently reinforces the established etiologic association between cumulative ultraviolet radiation exposure and the development of this neoplasm. However, the dual etiology of MCC, involving both UV exposure and Merkel cell polyomavirus (MCPyV) infection, complicates any simple preventive recommendations, as approximately 80% of MCC cases are MCPyV-positive, while 20% are MCPyV-negative and more strongly associated with UV damage [6,7].

From a histopathological and molecular perspective, MCC presents distinctive characteristics that facilitate its identification and differentiation from other cutaneous neoplasms. Neoplastic Merkel cells consistently express a characteristic immunohistochemical profile that includes specific epithelial markers such as AE1/AE3, epithelial membrane antigen, Ber-EP4, and CAM 5.2, as well as fundamental neuroendocrine markers including chromogranin, synaptophysin, calcitonin, and somatostatin [16]. However, the most specific and practically pathognomonic finding for MCC is the characteristic paranuclear staining pattern of cytokeratin 20, which is observed in more than 95% of MCC cases and serves as the most reliable diagnostic differentiator from other neuroendocrine neoplasms, particularly small cell lung carcinoma metastasizing to the skin, which typically shows diffuse cytoplasmic or negative CK20 staining [11,12].

The clinical evolution of MCC is characterized by exceptional aggressiveness, reflected in significantly elevated mortality rates, a phenomenon primarily attributed to the limited effectiveness of available therapeutic interventions for patients with established metastatic disease [17,18]. This therapeutic reality underscores the critical importance of early diagnosis and the implementation of aggressive multimodal treatment strategies in localized stages of the disease, where curative options maintain greater viability. The high propensity of MCC for early metastatic dissemination, both regional and systemic, requires a therapeutic approach that must be individualized and based on precise and comprehensive clinical staging.

Therapeutic strategies for the management of localized MCC have evolved toward a multimodal approach that emphasizes the importance of wide surgical excision with histologically negative margins, followed by the systematic evaluation of regional lymph node chains using lymphatic mapping and sentinel lymph node biopsy when clinically indicated [19]. Adjuvant radiotherapy has demonstrated efficacy in reducing local recurrence rates, which have historically been problematically high in this pathological entity, and constitutes an integral component of standard management in most cases.

The presented case paradigmatically exemplifies the complexities inherent in the management of MCC in advanced stages, where the presence of extensive lymph node metastases and the involvement of critical anatomical structures significantly limit the available curative therapeutic options. The palliative strategy implemented, centered on hypofractionated radiotherapy, demonstrated quantifiable clinical benefits with VAS pain reduction from 8/10 to 3/10 (62.5% improvement) and 40% tumor size reduction (10 cm → 6 cm), representing a realistic and appropriate therapeutic approach that prioritizes symptomatic control and the preservation of quality of life, considering both the extent of the disease and the individual patient characteristics, including advanced age and deteriorated functional status (ECOG 3).

This case precisely illustrates the devastating consequences of diagnostic delay, where the initial excision without histopathological analysis represented a missed opportunity that fundamentally altered the clinical course and available therapeutic options. The progression from potentially localized disease to T4N3M0 stage demonstrates how initial management deficiencies can transform a potentially curable condition (75% five-year survival) into advanced disease with significantly compromised prognosis (35% five-year survival) [15].

The evolution of MCC management toward more sophisticated and targeted therapies represents an active and promising area of research. The development of immunotherapeutic agents, particularly immune checkpoint inhibitors such as anti-PD-1 antibodies, has shown encouraging preliminary results in the treatment of advanced disease [17,19], although their availability and accessibility in different healthcare systems continue to be a significant challenge, especially in resource-limited environments. In this case, immunotherapy consideration was deferred due to the patient's deteriorated functional status (ECOG 3) and healthcare system limitations, despite the high Ki-67 index (80%) suggesting potential immunotherapy responsiveness.

A multidisciplinary approach emerges as a fundamental element in the optimal management of MCC, requiring effective coordination among specialists in dermatology, pathology, medical oncology, radiation oncology, and surgical oncology [14]. This interdisciplinary collaboration not only optimizes individualized therapeutic decisions but also facilitates the implementation of appropriate follow-up protocols and the early identification of recurrences or disease progression, crucial elements for maximizing therapeutic outcomes and overall survival.

This case illustrates several fundamental principles essential for optimal MCC management.

Mandatory histopathological analysis

Every cutaneous excision, regardless of clinical appearance or presumed benign nature, requires comprehensive histopathological evaluation. The absence of tissue analysis in this case directly contributed to the three-month diagnostic delay that transformed a potentially curable disease (75% five-year survival) into an advanced T4N3M0 stage (35% five-year survival).

Rapid growth is a red flag

Any cutaneous lesion demonstrating growth >1 cm within three months mandates immediate biopsy and specialist evaluation. This case exemplifies MCC's characteristic aggressive behavior with progression from 1 cm to 10 cm over four months, emphasizing the narrow therapeutic window available for early intervention.

Clinical-radiological discrepancy in MCC

Physical examination limitations in detecting deep lymphadenopathy are common in MCC, particularly in elderly patients with unfavorable neck anatomy. This case demonstrates how non-palpable clinical examination can coincide with extensive N3 nodal disease on imaging, reinforcing the mandatory role of cross-sectional imaging in MCC staging.

Multidisciplinary approach to diagnosis

Early involvement of specialized oncology teams is crucial for optimal outcomes. The complexity of MCC staging, treatment planning, and prognostic assessment requires coordinated expertise from dermatology, pathology, medical oncology, radiation oncology, and surgical oncology from the moment of diagnosis.

These learning points transform this case from an isolated clinical scenario into actionable knowledge that can prevent similar diagnostic delays and optimize patient outcomes in future MCC cases.

## Conclusions

This case demonstrates the severe consequences of delayed MCC diagnosis, with progression to advanced N3 stage and significantly compromised prognosis. The quantified palliative radiotherapy response was objectively documented with VAS pain score improvement from 8/10 to 3/10 (62.5% reduction), 40% tumor size reduction, and significant improvement in local symptom control, demonstrating the value of appropriate symptomatic management even in advanced disease.

Key clinical recommendations derived from this case are as follows. All cutaneous lesions, regardless of apparent benign nature, must undergo histopathological analysis following excision to prevent diagnostic delays that can transform curable disease into advanced stages. Rapidly growing lesions (>1 cm in <3 months) in elderly patients require immediate specialist evaluation and biopsy. Diagnostic delays in aggressive malignancies such as MCC can transform potentially curable disease (75% five-year survival) into advanced stages requiring palliative management (35% five-year survival). Early detection and timely management of localized disease remain the most crucial factors for improving long-term outcomes.

The clinical learning points emphasize the absolute necessity of histopathological analysis for all cutaneous excisions, the importance of high clinical suspicion for rapidly growing lesions in elderly patients, and the devastating consequences of diagnostic delays in aggressive malignancies. This case emphasizes the importance of standardized diagnostic protocols and multidisciplinary management to optimize outcomes in patients with this challenging disease, where initial management adequacy can determine the difference between cure and palliation.
